# Replicating Arabidopsis Model Leaf Surfaces for Phyllosphere Microbiology

**DOI:** 10.1038/s41598-019-50983-7

**Published:** 2019-10-08

**Authors:** Rebecca Soffe, Michal Bernach, Mitja N. P. Remus-Emsermann, Volker Nock

**Affiliations:** 1 Department of Electrical and Computer Engineering, University of Canterbury, Christchurch, New Zealand; 2 School of Biological Sciences, University of Canterbury, Christchurch, New Zealand

**Keywords:** Microbiology, Biomaterials

## Abstract

Artificial surfaces are commonly used in place of leaves in phyllosphere microbiology to study microbial behaviour on plant leaf surfaces. These surfaces enable a reductionist approach to be undertaken, to enable individual environmental factors influencing microorganisms to be studied. Commonly used artificial surfaces include nutrient agar, isolated leaf cuticles, and reconstituted leaf waxes. Recently, replica surfaces mimicking the complex topography of leaf surfaces for phyllosphere microbiology studies are appearing in literature. Replica leaf surfaces have been produced in agar, epoxy, polystyrene, and polydimethylsiloxane (PDMS). However, none of these protocols are suitable for replicating fragile leaves such as of the model plant *Arabidopsis thaliana*. This is of importance, as *A*. *thaliana* is a model system for molecular plant genetics, molecular plant biology, and microbial ecology. To overcome this limitation, we introduce a versatile replication protocol for replicating fragile leaf surfaces into PDMS. Here we demonstrate the capacity of our replication process using optical microscopy, atomic force microscopy (AFM), and contact angle measurements to compare living and PDMS replica *A*. *thaliana* leaf surfaces. To highlight the use of our replica leaf surfaces for phyllosphere microbiology, we visualise bacteria on the replica leaf surfaces in comparison to living leaf surfaces.

## Introduction

Researchers often turn to nature for inspiration in developing new and innovative technologies^1^. For instance, investigating the colour changing ability of chameleons for new surface properties or micro/unmanned air vehicles inspired from hummingbirds and dragonflies^2–4^. In addition, researchers also mimic nature to provide an insight into our natural world. This includes organs-on-a-chip for *in vitro* studies to minimise the use of animal surrogates, the development of microfluidic platforms for physiological studies, and developing artificial surfaces for phyllosphere microbiology^5–13^.

In phyllosphere microbiology, the study of microorganisms that reside on the leaves of plants, artificial surfaces are used to provide an insight into microbial behaviour in the phyllosphere^14^. The use of artificial surfaces, in place of a living leaf, enables a reductionist approach to investigate the impact of individual factors on microorganism function and viability, for example^13^. Artificial surfaces include: (1) flat surfaces, such as nutrient agar, and inert surfaces (*i*.*e*. stainless steel); and (2) microstructured surfaces, such as isolated leaf cuticles, leaf peels, reconstituted leaf waxes, and microfabricated surfaces^13,15–20^. Although these surfaces are suitable for their intended purpose, they do not fully represent the complex topography of plant leaves required for some phyllosphere microbiology studies^21,22^. This obvious shortcoming has led to the development of protocols utilizing double-casting approaches to produce leaf replicas in agar, epoxy, polystyrene, and polydimethylsiloxane (PDMS)^22–26^.

Traditionally, leaf surfaces have inspired researchers to replicate their superhydrophobic, superoleophobic, and superhydrophilic properties^2–4^. Such surfaces have applications in anti-reflection, anti-fouling, and anti-fogging coatings^4,26,27^. However, recently publications have started to appear which focus on replicating leaf surfaces for phyllosphere microbiology studies. This is attributed to increased interest into the role of microorganisms on plant health (*i*.*e*. foliar diseases)^13^. Especially for leafy greens, such as spinach, rhubarb, and parsley which are produced for human consumption. Which are exposed to a range of potential contamination sources during production on a farm, which can lead to unwanted microorganisms introduced into the phyllosphere. Furthermore, leafy greens are often consumed either raw or without minimal processing; thus, unwanted contamination is neither removed nor killed^10–13,28–31^. On the rare occasion, contamination can lead to outbreaks that can result in severe illnesses^32–34^. Hence, new studies are imperative for increased understanding of phyllosphere microbiology, which will enable mitigation strategies to be developed^34,35^. This is of considerable importance with the projected changes to our dietary consumptions due to climate change, which will see humans transitioning from a meat-based diet to a plant-based diet^36,37^.

Existing examples for replica leaves in phyllosphere microbiology include the use of epoxy-based replication to investigate the influence of cuticular folds on the behaviour of Colorado potato beetles (*Leptinotarsa decemlineata*)^38^. In another study, Zhang *et al*. replicated spinach leaf surfaces to produce replicas in agarose and PDMS. In this paper they investigated the behaviour of *Escherichia coli* on flat agarose and agarose replica spinach leaf surfaces^23^. Recently we performed an extensive investigation to determine a suitable replica leaf material for phyllosphere microbiology^20^. In this investigation we compared the resolution and degradation characteristics of patterned agarose, PDMS, and gelatin, and conducted contract angle and bacterial survival measurements. Our results revealed that PDMS is the most suitable replica leaf replica material, due to the high fidelity and favourable degradation characteristics achieved with PDMS, while exhibiting comparable hydrophobicity and bacterial survival characteristics observed on generic isolated leaf cuitcles^20^.

To date reported protocols have used plants grown in sub-optimal conditions (*i*.*e*. botanical gardens), such as inconsistent water supply and uncontrolled temperature and light conditions^24^. However, for phyllosphere microbiology, plants are often grown in optimal conditions in either soil or media culture in growth chambers. Growing plants in optimal conditions, especially in media culture results in leaves with a high water content and a thin cuticular wax layer^39,40^. Imprints produced in polyvinylsiloxane do not allow for degassing to maximise resolution of small microstructures - due to the quick curing time^24,38^. Alternatively, light curable polymers produce undesirable chemical and ultraviolet (UV) light exposure to the plant leaf^41^.

Conversely, leaf imprints produced in PDMS are not exposed to undesirable chemical or UV exposure, and reported protocols for replicating leaves have shown good resolution^23,26,42^. However, these existing protocols have some major drawbacks, which are more apparent when imprinting leaves from plants grown in optimal conditions. The three major drawbacks we identified were: (1) the leaves being exposed to excessive heat during curing, which resulted in the leaves shrivelling during curing, and subsequent loss of fidelity of the imprint; (2) the leaf samples retained too much water, which resulted in the PDMS not curing over the entire leaf surface; and (3) some leaf residue remained affixed to the imprints^23,26,42,43^. All of these drawbacks affected the quality of the imprint and subsequent replica^41^. Furthermore, we selected *Arabidopsis thaliana* as our model replica leaf, as it is the best model system for molecular plant genetics and molecular plant biology, and it is a well-established model system for microbial ecology^10,44,45^. However, the *A*. *thaliana* leaves are inherently fragile which presents an additional challenge to reproduce the microstructures of *A*.*thaliana* into PDMS with high fidelity.

As a result, we developed a versatile protocol to overcome these limitations and maximise the fidelity of the leaf imprint, and subsequent replica leaf. For the PDMS imprint, we use a base to curing agent ratio of 20:1, whereas published procedures use a PDMS ratio of 10:1 for both the imprint and replica^23,42^. The prevalent use of a ratio of 10:1 could be due to this ratio being commonly used for PDMS-based microfluidic channels, and in other double-casting applications, such as bioimprinting^46–48^. Prior to casting the leaf imprint we briefly dried *A*. *thaliana* samples to remove any surface moisture from the leaves. This was done to ensure the PDMS completely cures on the surface. To avoid excessive heating, the PDMS imprints were cured at 45 °C for 20 h. To minimise any leaf residue affecting the resolution of the subsequent replica leaf, the PDMS imprints were placed in a leaf digestion solution to remove any leaf residue. Following this, to produce the replica leaves a conventional double-casting protocol was employed, through the application of an anti-adhesion coating to the leaf imprint prior to casting^47–50^. This enable PDMS replica leaves to be produced at a traditional ratio of 10:1 and readily peeled off the imprint. To highlight the capacity of our replication protocol we used optical microscopy, atomic force microscopy (AFM), and contact angle measurements to compare replica and living *A*. *thaliana* leaf surfaces. Finally, we demonstrate the application of replicated *A*. *thaliana* leaf surfaces for phyllosphere microbiology by introducing bacteria onto their surfaces.

## Methods and Materials

### Growth protocol for *a*. *thaliana* plants grown in soil

Plastic plant pots (70 by 70 by 90 mm) were filled with potting mix containing 9 month-controlled release fertiliser (Canterbury Landscape, New Zealand), 20 mm below the top of the pots. A 2 g scoop of No Insects Lawngard Prills (KiwiCare, New Zealand) was added to the soil. The last 20 mm of the plant pots were filled with potting mix sieved with five mm mesh. *A*. *thaliana* ecotype Columbia 0 (Col-0) wild type seeds were suspended in water, and then placed on top of the soil in the plant pot. Following this, the plant pots were placed on a raised tray with holes, to allow the water to be gradually absorbed by the soil in the plant pots. This tray was then placed in a container with a clear cover containing holes. The plants were grown under long day conditions as follows: 16 hours of daylight at 21 °C and eight hours of darkness at 18 °C, and the plants were watered weekly from initial potting. Seedlings were trimmed to ensure that only one seedling remained. Furthermore, the cover of the container was removed after three weeks.

Two days prior to replication of the leaf surfaces, the plants were removed from the growth chamber and placed on a bench in a climate-controlled laboratory – relative humidity (30%) and temperature (25 °C). This was done to reduce the internal water content of the plants, which reduced the amount of moisture being realised from the leaf samples when curing PDMS.

### Growth protocol for *a*. *thaliana* plants grown on culture media

Initially the *A*. *thaliana* seeds were sterilised using a standard protocol, as follows: (1) The desired amount of *A*. *thaliana* Columbia (Col-0) wild type seeds was placed in a 1.5 mL Eppendorf tube; (2) Then 1 mL of 50% bleach (Dynawhite, Jasol, New Zealand) was added to the Eppendorf tube; (3) After five minutes, the bleach was removed from Eppendorf tube; (4) Then 1 mL of 70% ethanol (Anchor) was added to the Eppendorf tube; (5) After one minute the ethanol was removed, and the seeds were washed five times with 1 mL of sterile deionised water^51^. After sterilisation, the seeds were stratified at 4 °C in sterile deionised water for at least two days in the dark.

Glass jars (741 Mold Jar, Weck Jars, Germany) were used to grow the *A*. *thaliana* plants on culture media. Prior to adding the seeds and culture media, the glass gars were sterilised by autoclaving. Then 65 mL of Murashige and Skoog medium (pH 5.8, M0222, Duchefa Biochemie, Netherlands) with 0.6% w/v plant agar (P1001, Duchefa Biochemie, Netherlands), and 1% w/v of Sucrose (Product info, company) was added to the jars. The open jars were then placed in laminar flow for an hour, to cure the agar and dry the media. To further minimise the potential occurrence of contamination, the media filled jars were placed under laminar flow and sterilised with UV light for 20 minutes. Once sterile the *A*. *thaliana* seeds were placed on the surface of the culture media and the lid was secured on the jar. The jars were then placed inside a growth chamber (Contherm Precision Environmental Chamber, Contherm Scientific, New Zealand) in long day growth conditions (16 hours of daylight at 21 °C and eight hours of darkness at 18 °C).

### Preparation of *a*. *thaliana* leaf samples

**Figure 1:**
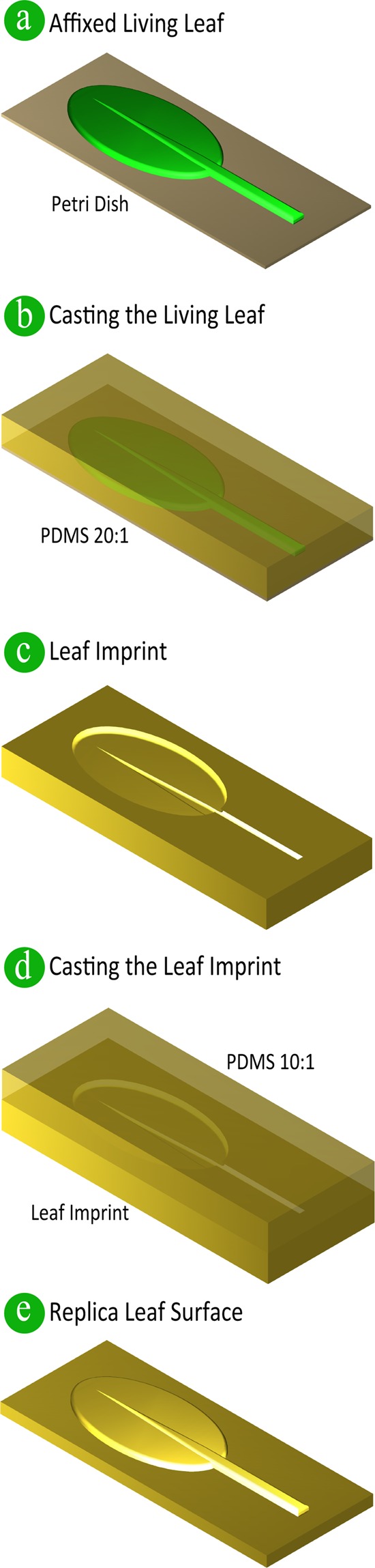
Schematic of the Replication Protocol for Replica Leaf Surfaces in PDMS. (**a**) Affix a living leaf sample to a petri dish with double-sided tape. (**b**) PDMS with a ratio of 20:1 is then poured on to the affixed leaf, with the PDMS degassed and cured. (**c**) The leaf imprint is then carefully peeled off. Prior to pouring the leaf imprint with PDMS for double-casting, an anti-adhesion coating is applied. (**d**) PDMS with a ratio of 10:1 is poured onto the imprint, with the PDMS being degassed and cured. (**e**) Once cured the leaf replica is peeled carefully from the leaf imprint.

Double-sided mounting tape (Orabond 1397PP, ORAFOL Europe GmbH, Germany) was placed on to the bottom of a polystyrene petri dish. Care was taken to ensure that the tape was sufficiently flattened, to minimise affecting the quality of the imprint and subsequent replica. Note that the tape used should be larger than the size of the leaf, rather than using multiple pieces of tape. The use of multiple pieces of tape caused uneven surfaces and bubbles to form in the gaps between the pieces of tape. Leaves were taken from the *A*. *thaliana* plants, rinsed with deionised water to remove any contaminants, and promptly dried with low pressure dry nitrogen. The leaves were either kept whole, or cut into smaller samples, and placed on the double-sided tape. Following this, the leaves were carefully flattened by pressing gently on the leaves with softened Parafilm “M” (Bemis, USA) (Fig. 1a). The samples were then placed in an automatic desiccator (Secador 2.0, SP Scienceware Bel-Art, USA) or a vacuum desiccator (Z119016, Sigma-Aldrich) until no residual moisture was observed on the surface of the leaf samples. This process was selected to minimise damage to the microstructures of the leaf samples, and to remove any excess moisture from the top surface of the leaves which prevented the PDMS from setting^52^.

### Leaf imprint protocol

The PDMS (Sylgard 184, Dow Corning) was prepared at a ratio of 20:1 w/w, base to curing agent, thoroughly mixed, and then degassed in a vacuum desiccator (Z119016, Sigma-Aldrich) until no bubbles remained^43^. The PDMS was then promptly poured onto the leaves affixed to the petri dish (Fig. 1b). After pouring, the PDMS was degassed for an hour, or slightly longer if any bubbles remained. The petri dish was then placed in an oven (Contherm Scientific, New Zealand) at 45 °C for 20 hours – to cure the PDMS. Curing for a longer duration resulted in an increase in leaf residue remaining on the imprint. We conjectured that this was due to the degradation of the leaf cuticle^43^. Once the PDMS was cured, the petri dish was removed from the oven and left to cool to room temperature to enable the imprint to be carefully peeled from the leaf sample (Fig. 1c).

### Removing leaf residue from the PDMS imprint

To remove any leaf residue, a digestion solution comprising of 3.5% w/v sodium hydroxide (NaOH, S5881, Sigma-Aldrich) and 2.5% w/v sodium carbonate (Na_2_CO_3_, 222321, Sigma-Aldrich), in deionised water was used^53^. The digestion solution was placed on a hotplate at 160 °C and stirred at low angular speed (80 to 120 rpm, depending on the size of the glassware and magnetic stirrer used) (SP88857105, Cimarec+, Thermo Scientific), until the sodium hydroxide and sodium carbonate were completely dissolved – approximately 15 minutes. The PDMS leaf imprint was placed into the digestion solution for 20 minutes. Once removed from the digestion solution, the PDMS leaf imprint was promptly rinsed thoroughly with deionised water. Any stubborn residue was carefully removed with tweezers with the PDMS leaf imprint submerged in deionised water (this was observed to occasionally around the edges of the PDMS leaf imprint). The PDMS leaf imprint was then placed in fresh deionised water for 20 minutes to remove any digestive solution residue. Finally, the imprint was rinsed thoroughly with deionised water and dried with dry nitrogen.

### Anti-adhesion coating

An anti-adhesion coating for PDMS double-casting can be produced in one of two ways: (1) Treatment with hydroxypropylmethylcellulose (HPMC, H8384, Sigma-Aldrich). In this case the leaf imprint was treated by immersion in 0.3% w/v HPMC in phosphate buffer saline (PBS, P4417, Sigma-Aldrich) for 10 minutes for anti-adhesion. The imprint was then rinsed with deionised water, and subsequently dried with nitrogen^47,48^. Alternatively, (2) treatment with trichloro(1H, 1H, 2H, 2H-perfluorooctyl)silane (FDTS, 448931, Sigma-Aldrich), can be undertaken. The imprint was initially treated with oxygen plasma for 60 s to produce a hydrophilic surface (15 W, pulse ratio: 50, 3 sccm O_2_; PIE Scientific Tergeo Plasma Cleaner, USA). The imprint was then placed in a vacuum desiccator (Z119016, Sigma-Aldrich) alongside an open glass bottle containing a small droplet of FDTS and placed under vacuum for an hour^49,50^.

### Replica leaf in PDMS protocol

For the PDMS replica leaf, the base and curing agent were thoroughly mixed together at a ratio of 10:1 w/w (base to curing agent). Once mixed the PDMS was degassed in a vacuum desiccator (Z119016, Sigma-Aldrich) until no bubbles remained. The PDMS was subsequently poured onto the anti-adhesion coated leaf imprint and degassed for an hour (Fig. 1d). The leaf imprint and PDMS were then placed on a hotplate for two hours at 80 °C to cure. Finally, the PDMS replica leaf was carefully peeled off the leaf imprint, once the PDMS had cooled to room temperature (Fig. 1e).

### Atomic force microscopy (AFM) imaging

All AFM images were taken using a MFP-3D Origin (Asylum Research - Oxford Instruments, USA), equipped with either TAP150Al-G (*A*. *thaliana*) or TAP300-G (PDMS imprint and replica) tips (BudgetSensors, USA) operating in tapping mode. All AFM scans were analysed using Gwyddion (ver. 2.49).

### Contact angle measurements

Contact angle measurements were obtained using a CAM200 (KSV Instruments Ltd, Finland), integrated with KSV CAM Optical Contact Angle and Pendant Drop Surface Tension Software (ver. 4.01, KSV Instruments Ltd, Finland). Deionized water was used to determine the surface energy of the *A*. *thaliana* and PDMS leaf replica. Five samples were measured for both *A*. *thaliana* and PDMS replica leaves. In all cases the samples were obtained using a cork borer (Usbeck, Germany) with a diameter of 11.5 mm. Water droplets with a volume less than 60 µL were recorded.

Results are presented as mean ± SEM (standard error of the mean). For statistical analysis, two-way ANOVA was performed using GraphPad Prism 7 (GraphPad Software, USA). P values less than 0.05 were considered significant (*P < 0.05, **P < 0.01, ***P < 0.001, and ****P < 0.0001).

### Bacteria culture protocol

Two bacterial strains were used in our investigations: *Pantoea agglomerans* 299 R::MRE-Tn7-145 and *Sphingomonas melonis* Fr1::MRE-Tn5-145. Both bacterial strains were grown overnight on nutrient agar plates (13 gL^−1^ Lysogeny broth and 15 gL^−1^ bacteriological Agar, Oxoid) containing 20 mgL^−1^ of gentamicin (AG Scientific) at 30 °C^54^. The bacteria was then harvested using a sterile inoculation loop and was suspended in 5 mL of sterile phosphate buffer saline (PBS pH 7.4, P4417, Sigma-Aldrich). The bacteria was then washed by centrifugation at 1150 RCF for five minutes at 10 °C. The supernatant was discarded, and the bacteria was suspended in fresh phosphate buffer, and washed for a second time – following the aforementioned process. After washing for a second time the bacteria were suspended in PBS to an OD_600 nm_ of 0.2.

### Bacteria visualisation protocol

Living *A*. *thaliana* leaves and PDMS replica leaves require different sample preparation for bacteria visualisation studies, undertaken using microscopy. The *A*. *thaliana* leaf samples were taken from mature *A*. *thaliana plants*, immediately prior to inoculation of bacteria and subsequent microscopy. The abaxial leaf samples were first washed with deionised water to remove any contaminants. The leaf samples were then dried with either filtered compressed air or nitrogen. For microscopy, the leaf samples were affixed to glass microscopy slides with double-sided tape.

Whereas, the PDMS replica leaves were sterilised for 15 minutes using UV sterilisation. Once sterilised, the replica leaves were placed in vacuum desiccator (Z119016, Sigma-Aldrich) for two hours. Following this, the replicas were placed in PBS overnight at room temperature. Prior to the inoculation with bacteria, the PDMS replicas were dried with either filtered compressed air or nitrogen.

Prior to microscopy two aliquots of 200 µL of bacterial solution were inoculated onto the living *A*. *thaliana* or PDMS leaf replica samples using an airbrush (KKmoon T-180 Airbrush, China) at 1 × 10^5^ Pa^55^. Differential interference contrast (DIC) and fluorescent images were then obtained using a Zeiss Axiolmager M1 fluorescent widefield microscope equipped with a 43HE Zeiss filter set (Zeiss, Germany). Images were acquired using a 20× objective (Zeiss, Germany), and an Axiocam 506 camera (Zeiss, Germany) controlled by Zeiss Zen software (ver. 2.3). The DIC and fluorescent images were obtained using DIC 2 (1.5 ms exposure time) and the red fluorescent channel (850 ms exposure time), respectively. Images were then processed using Fiji (ver. 1.52 h), and the extended depth of focus plug-in was utilised to process the DIC channel^56^.

## Results and Discussion

### Replication protocol

**Figure 2:**
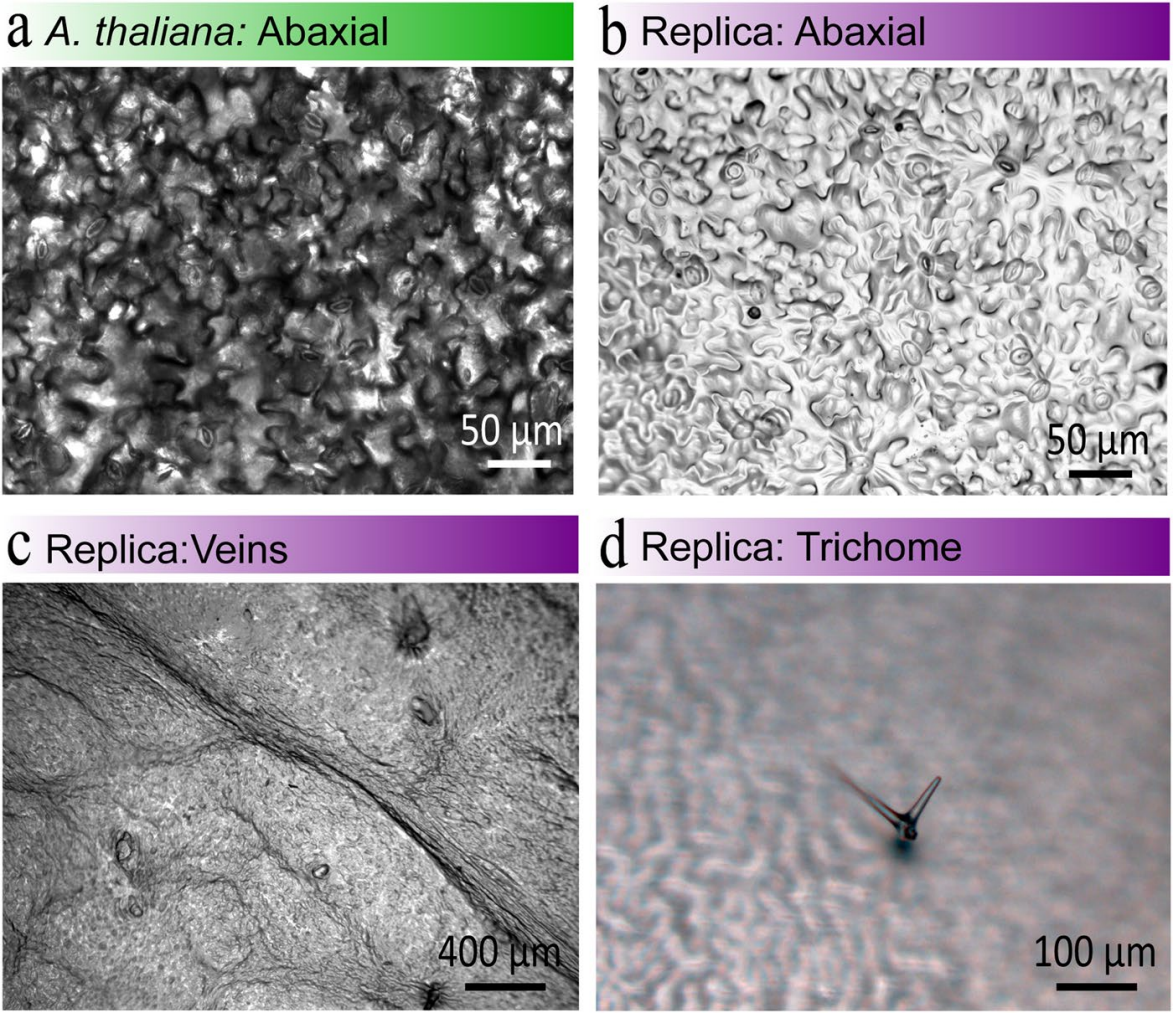
Optical Microscopy Images of *A*. *thaliana* Living and Replica Leaves from Plants Grown in Soil in Optimal Conditions. (**a**) A living leaf. (**b**–**d**) Replica leaf surfaces, highlighting the different structural aspects of the leaf. Note: abaxial surface is the lower leaf surface, and trichomes are leaf hairs. See Fig. S1 for optical microscopy images from a replica leaf surface from a plant grown in culture media.

The replication protocol displayed in Fig. 1 was used to replicate the leaves of *A*. *thaliana* plants, which is a well-established model system for microbial ecology^44^. For our replication protocol we used leaf samples from mature *A*. *thaliana* plants that had been grown for four to six weeks. We successfully replicated the surface topography of leaves from plants grown in optimal conditions in soil (Fig. 2) or media culture (Fig. S1**)**. Although, growing plants in optimal conditions in either soil or culture media does not influence the topography of the leaf surface, our protocol needed to work for both soil and culture media grown plants to enable microbiologists to replica their own plant leaves for their own phyllosphere studies. This is opposed to plants grown in soil, which are used for studies that focus on the plant. Conversely, if the plants are required to be axenic (free from living microorganism) for investigating microorganism communities, the plants are grown in sterile culture media^57^. In the following paragraphs we discuss the details of our protocol and highlight considerations made for leaves from plants grown in optimal conditions (reduced cuticular wax layer and increased water content) and fragile leaves, for example, *A*. *thaliana* which we use as our model plant.

Initially, to produce the PDMS leaf imprints **(**Fig. 1b**)** we followed standard PDMS double-casting protocols, which have also been used to produce leaf replicas of leaves from various plants (*i*.*e*. spinach, trembling aspen, lotus)^42,47–49,58^. However, the high temperatures (~80 °C) used to cure the PDMS imprint, resulted in the *A*. *thaliana* leaf samples degrading, and having a dramatic effect on the imprint quality. An alternative protocol proposed by Wu *et al*. imprinted the leaf topography for producing microfluidic channels representative of the leaf nervature in PDMS^43^. In this protocol, the PDMS was prepared at a ratio of 20:1 (base to curing agent), and cured in an oven for 24 hours at 45 °C. However, despite the lower stiffness and temperature used, we repeatedly observed sections of PDMS in contact with the *A*. *thaliana* leaf samples not curing. We conjectured this was due to the high moisture content of the leaf samples. As a result, we developed a drying process to minimise the surface moisture content of the leaves, while ensuring fidelity of the samples. This was undertaken by placing the *A*. *thaliana* samples affixed to the polystyrene petri dish substrates into either an auto-desiccator cabinet or a vacuum desiccator. The samples were kept in the desiccator until the surface moisture of the leaves had visually reduced. This process enabled control over the leaf drying and minimised the degradation of the leaf sample. As an aside we also observed that drying the leaf sample prior to affixing the sample to the petri dish resulted in the leaf shrivelling, which rendered the sample unusable. In addition, we decreased the curing time to 20 hours to minimise degradation of the leaf cuticular waxes. The degradation of the leaf cuticular waxes has been conjectured to result in the leaf remaining attached to the imprint, after peeling the imprint away from the leaf sample^43^.

Periodically we observed leaf residue around the edges of a leaf imprint, which impacted the fidelity of the subsequent replica leaf. To that extent we looked for an approach that would not damage the PDMS imprint, whilst effectively removing any leaf residue. A digestion solution provided an easy approach, as they are well-established and effective at deteoriating leaf tissues. We selected a digestion solution of sodium hydroxide and sodium carbonate, as this method was quick in comparison to enzyme digestion protocols. No adverse effects were observed on the PDMS imprint using this digestion solution (Supplementary Information S2). Furthermore, the resulting PDMS leaf imprint was able to be reused until the PDMS imprint degraded. We regularly used imprints over 10 times and observed no apparent changes in the fidelity of the subsequent replica leaf surfaces.

We recently undertook an in-depth analysis to determine a suitable replica leaf surface material for phyllosphere microbiology. From this we concluded that PDMS is the most suitable material for producing replica surfaces, in comparison to agarose and gelatin, due to: resolution characteristics, material stability in environmental conditions, hydrophobicity and bacterial survivable comparable to that of an isolated leaf cuticle^20^. Therefore, we developed our replication protocol to produce a PDMS replica leaf. To produce the PDMS replica leaf surface, the imprint was treated with an anti-adhesion coating. Two different approaches were utilised, where the surface was treated with HPMC or FDTS^47–50^. We found that treatment with FDTS allowed the PDMS leaf replica to be peeled off the leaf imprint significantly easier than those treated with the HPMC solution. We conjecture this is due to a smaller adhesive force of imprints coated with FDTS rather than HPMC^50^. This is more important for leaf surfaces with an abundance of trichomes, which are inherently fragile and can readily breakoff when the replica leaf is being peeled away from the leaf imprint. However, if one does not have access to a plasma asher/cleaner that is required to produce oxygen plasma for FDTS treatment, then producing a PDMS replica using HPMC as an anti-adhesion coating will suffice. We were unable to observe any apparent differences in replica *A*. *thaliana* abaxial (lower) surfaces produced by either method – which do not exhibit large numbers of trichomes.

Due to the complex nature and large aspect-ratio of trichomes, the fabrication protocol was improved to increase trichome yield. Trichomes similarly to other microstructures (stomata and grooves) influence the colonisation behaviour of bacteria, so their ability to be replicated is important for phyllosphere microbiology studies^14^. This was achieved by degassing the leaf imprint at various stages of the fabrication protocol for two hours each time, which assisted in the passive filling of complex microstructures, such as trichomes^59^. For instance, degassing occurred prior to placing the leaf imprint in the digestion solution. As *A*. *thaliana*, trichomes are predominantly found on the adaxial (upper) surface of the leaf, these additional steps were only undertaken when replicating the adaxial surface. However, the overall yield for complete trichomes is relatively low, and further improvements need to be made in this area.

The PDMS leaf replica surfaces were produced using PDMS mixed at a ratio of 10:1 (base to curing agent) and cured for two hours on a hot plate **(**Fig. 1d**)**. Once cooled to room temperature, the replica was carefully peeled off the leaf imprint **(**Fig. 1e**)**, and the fidelity of the PDMS leaf replicas were examined using optical microscopy and atomic force microscopy (AFM).

### Optical imaging

To examine the fidelity of our *A*. *thaliana* PDMS replica leaf surfaces, we initially used optical microscopy (Fig. 2 and S1). The topography of leaf surfaces vary considerably and different microstructures can have different structures. Microstructures that can be found on the surface of *A*. *thaliana* leaves include stomata, trichomes, and grooves^60,61^. Stomata are pores that control transpiration, and enable gas exchange to occur between the atmosphere and the leaf. For instance, stomata enable carbon dioxide to enter the leaf, which is important for photosynthesis to occur^12,62^. Whereas, trichomes minimise water loss from the leaf surface, regulate the temperature of the leaf, reduce the effects of UV radiation, and/or secrete metabolites to deter herbivores and inhibit pathogen development^12,63^.

For our PDMS replica leaves we were primarily focused on replicating the abaxial (lower) surface of *A*. *thaliana* leaves, due to bacteria being more abundant on the abaxial surface (Fig. 2a,b)^44,64,65^. Thus our work naturally focussed on the fidelity of the replicated stomata and grooves – the most abundant microstructures on the abaxial surface of *A*. *thaliana* leaves (Fig. 2a). The length and width of stomata on the replica *A*. *thaliana* leaves were measured to be 14.0 ± 1.2 µm and 9.5 ± 1.1 µm, respectively (Fig. 2b). Which is comparable to the length and width of stomata on living *A*. *thaliana* leaves, which were measured to be 16.8 ± 2.5 µm and 9.0 ± 1.5 µm, respectively (Fig. 2a). As an aside, due to the optical transparency of PDMS we found imaging the replica leaf surface (Fig. 2b) easier than the surface of a living leaf (Fig. 2a). As the transparency of a living leaf is hindered by the leaf tissues, which results in a darker image compared to an image of a replica leaf. Furthermore, the roughness of the replica leaf surface can be observed in Fig. 2c. This highlights the ability of our replication protocol to effectively replicate the roughness of the surface, whilst simultaneously retaining ranges of microstructure from stomata and grooves (Fig. 2b) to larger features such as veins, which are apparent in Fig. 2c.

In addition, a PDMS replica trichome is presented in Fig. 2d, highlights the potential of our approach for replicating trichomes found on *A*. *thaliana* leaves. Previously, replicating trichomes has only been achieved using polyvinylsiloxane imprints in combination with epoxy replicas^22,24^. Consequently, a potential future improvement for replicating *A*. *thaliana* trichomes might be an approach using polyvinylsiloxane imprints to produce PDMS replica leaves. However, negotiating the quick curing time of polyvinylsiloxane is necessary for producing high quality leaf imprints^24,38^. The use of PDMS for replica leaves is more favourable than epoxies due to the biocompatibility and optical transparency of PDMS; in addition to the ease of changing the surface hydrophobicity of PDMS for attachment studies, and the ability to add fillers to PDMS for nutrient studies^66,67^. Furthermore, the surface energy and bacterial viability on PDMS replica leaf surfaces are comparable to leaf surfaces^20^.

### AFM imaging

**Figure 3:**
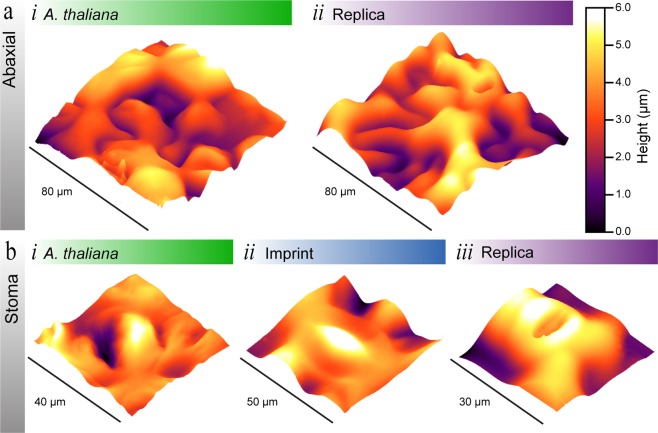
AFM Characterization of *A*. *thaliana* Leaf Surfaces. (**a**) AFM images of the abaxial surface of (*i*) living and (*ii*) replica *A*. *thaliana* leaves. (**b**) AFM images of stoma found on the abaxial surface of (*i*) living, (*ii*) imprint, and (*iii*) replica *A*. *thaliana* leaves. A larger area scan of the (*ii*) stoma imprint is displayed in Fig. S2.

To further investigate the suitability of our replication protocol for producing leaf replicas in PDMS we used AFM imaging **(**Fig. 3**)**. Due to the nature of AFM imaging the leaf surfaces with AFM presented some challenges: (1) The extent of surface roughness of the leaf surface, as an AFM imaging is optimised for measuring the topography of films with nanoscale topography - in addition to nanomechanical and electrochemical characterisation. To compensate for the roughness of the leaf surfaces, we used an MFP-3D Origin AFM with a large working height range of 15 µm. (2) Obtaining high resolution images using AFM can take anywhere from a few minutes to several hours depending on the sample. This was problematic when imaging the *A*. *thaliana* living leaf samples, as the leaves readily degraded during imaging. Thus, the degradation effected the resolution of the AFM images, and increased the probability of the cantilever tip losing contact. Using an MFP-3D Origin AFM enabled us to minimise any potential degradation of the living leaf due to images being obtained within 30 minutes of being prepared. In addition, all leaf samples were carefully flattered and affixed to a glass substrate with double-sided tape to minimise any potential damaged to the leaf microstructures. (3) The softness of the *A*. *thaliana* living leaf, PDMS imprint, and PDMS replica, can also influence the quality of the AFM image due to the cantilever not sufficiently tracking the surface of the sample. We maximised surface tracking by selecting an appropriate cantilever for each sample. (4) Finally, the A. thaliana leaf surface minimally reflects the microscope light which resulted in limited visibility when selecting an area for imaging. Which made it a challenge for us to find stomata and flat areas suitable for imaging – which currently rules out registered AFM.

As our interest was focused on replicating the abaxial surface, we focused on investigating the fidelity of grooves and stomata, which can be seen in Fig. 3. The large area topographical AFM images in Fig. 3a of the *A*. *thaliana* living leaf and the PDMS replica leaf, display the roughness and variability of the leaf topography. An important microstructure on the abaxial surface of *A*. *thaliana* are stomata, which can be seen in Fig. 3b. The acquired AFM images of a stoma from a living leaf, leaf imprint, and replica leaf have comparable morphology and dimensions. The slight variances of the size and positioning of the stoma can be attributed to scanning different stoma. Therefore, the acquired AFM images and optical images indicate that the replication protocol is suitable for reproducing *A*. *thaliana* leaf replicas in PDMS with high fidelity.

### Contact angle measurements

The cuticle, a wavy layer, which covers plant leaves and protects the plant from the external environment^68^. In particular, the cuticle prevents water, ion, and nutrient loss, and protects against pathogenic attacks^69^. An important property of the cuticle is its surface energy, and in particular its hydrophobicity. In phyllosphere microbiology the surface hydrophobicity is important, as the presence of water on the leaf surface influences resources availability and microorganism colonisation patterns^70^. In addition, microorganism attachment processes are influenced by the hydrophobicity of the leaf cuticle, and attachment can be achieved by adapting to enable attachment, or by forming biofilms^13,71^.

The surface energy of a surface can be classified as either hydrophilic, hydrophobic, or superhydrophobic, when the contact angle of water is <90°, >90°, and >150°, respectively. The measured contact angle of leaf cuticles/surfaces can vary considerably, from hydrophilic to superhydrophobic depending on the plant species^26,72,73^. We used deionised water to conduct the contact angle measurements and recorded droplets with a volume less than 60 µL.

**Figure 4:**
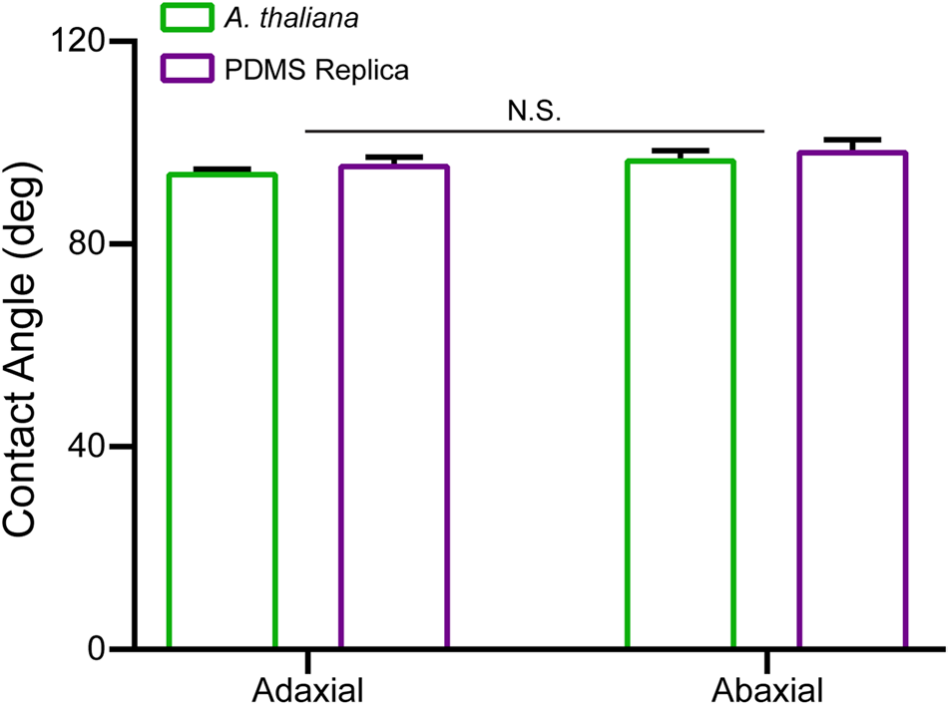
Contact Angle of Living and PDMS Replica A. *thaliana* Leaf Surfaces. Contact angle measurements of living and replica leaf samples are shown for both the adaxial (upper) and abaxial (lower) surfaces of A. *thaliana*. Results are presented as mean ± SEM (standard error of the mean). N.S. indicates no significant difference between the measurements.

Contact angles were obtained for the adaxial (upper) and abaxial (lower) surfaces of leaf samples from mature *A*. *thaliana* plants grown in soil **(**Fig. 4**)**. This was undertaken as some plants have differing hydrophobic properties between the adaxial and abaxial surfaces^72,73^. In the case of *A*. *thaliana*, no significant difference (N = 5) was observed between the adaxial and abaxial surfaces **(**Fig. 4**)**. A mean contact angle of 97 ± 1° for *A*. *thaliana* was measured; thus, indicating that the surface of *A*. *thaliana* leaves are hydrophobic.

In addition, contact angle measurements were obtained for *A*. *thaliana* PDMS replica adaxial and abaxial leaf surfaces **(**Fig. 4**)**. No significant difference (N = 5) was observed between the PDMS replica adaxial and abaxial leaf surfaces. A mean contact angle of 99 ± 1° for the PDMS replica leaves was measured; thus, indicating that the surface of the PDMS replica leaves are also hydrophobic. Furthermore, no significant difference in the contact angle was observed between the *A*. *thaliana* and PDMS replicas leaf surfaces.

One advantage of PDMS replica surfaces is that they provide the ability to examine the influence of hydrophobicity and the role this has on attachment studies. As the surface hydrophobicity of PDMS can be temporarily modified with the use of oxygen plasma. This duration can be extended through the use of polyvinylpyrrolidone (PVP) treatment. Short oxygen plasma and PVP treatments are not considered harmful to microorganisms – note the microorganisms would be inoculated to the surface after treatment^67^. This enables more extensive and controlled attachment studies to be undertaken using a PDMS replica leaf surface, compared to living leaf samples and other artificial leaf surfaces. Which is important, as attachment studies have been highlighted as an area in phyllosphere microbiology which requires more extensive studies to be undertaken^13^.

### Bacterial visualisation studies

**Figure 5:**
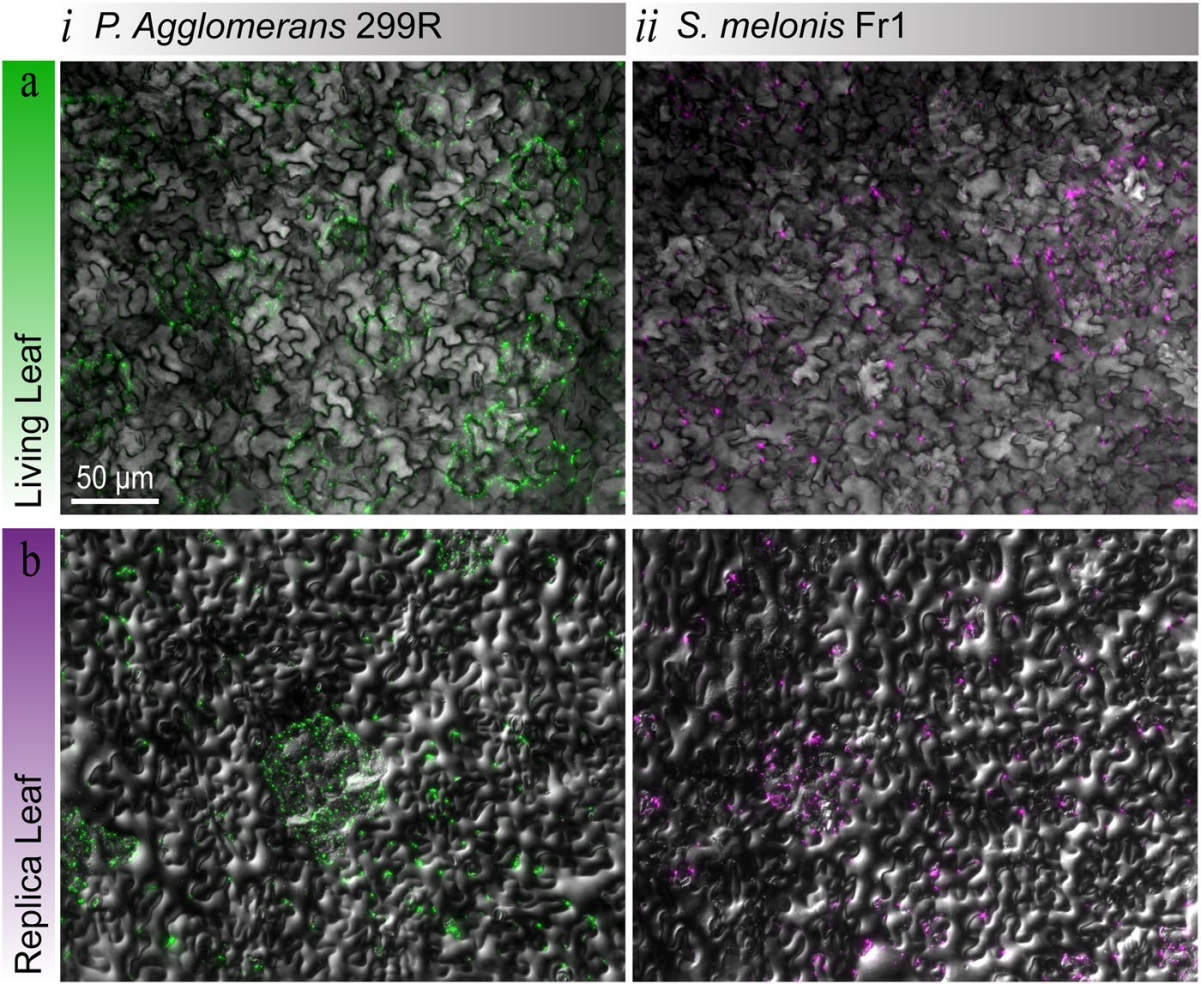
Bacterial Distribution on Abaxial Leaf Surfaces. The bacteria (*i*) *P*. *agglomerans* 299 R::MRE-Tn7-145 (green) and (*ii*) *S*. *melonis* Fr1::MRE-Tn5-145 (magenta) were visualised on (**a**) living and (**b**) replica abaxial leaf surfaces. See Fig. S3 for P. agglomerans 299 R::MRE-Tn7-145 visualisation of living and replica surfaces with an OD_600 nm_ of 0.7.

To examine the suitability of the PDMS replica leaf for phyllosphere microbiology, we used the bacterium *Pantoea agglomerans* 299 R and *Sphingomonas melonis* Fr1 as our model microorganisms **(**Fig. 5 and Fig. S3**)**. The bacterium *P*. *agglomerans* 299 and *S*. *melonis Fr1* were previously isolated from a Bartlett pear tree leaf, and Arabidopsis leaf material, respectively^54,74,75^. We selected *P*. *agglomerans* 299 and *S*. *melonis* as our model microorganisms, as they are: (1) model microorganisms for leaf colonisation studies; (2) well characterised and fully sequenced; and (3) are genetically amendable (able to produce mutants and bioreporters)^54,75^. The bacteria were previously modified using a genomic Tn7 or Tn5 transposon insertion carrying an mScarlet-1 fluorescent protein due to its high brightness, with the microorganisms identified as *P*. *agglomerans* 299 R::MRE-Tn7-145 and *S*. *melonis* Fr1::MRE-Tn5-145^76^.

The bacteria distributions on the living *A*. *thaliana* **(**Fig. 5a**)** and PDMS replica **(**Fig. 5b**)** abaxial surfaces, were influenced by the distribution of the bacteria suspension droplets. As discussed previously, minimal wetting was observed on both living *A*. *thaliana* and PDMS replica leaf surfaces **(**Fig. 4**)**. Which indicated that the resulting shape of the dispersed droplets were influenced by the leaf microstructures on the abaxial surface (grooves and stomata). We observed that bacteria were predominantly confined to the droplets, which supports the assumption that there was no residual surface moisture on the living *A*. *thaliana* or the PDMS replica leaf. Furthermore, we repeatedly observed more bacteria at the edge of the droplet interface in direct contact with the surface of either living or PDMS replica *A*. *thaliana* leaf surfaces. Thus indicating, that the distribution of bacteria observed on the PDMS replica leaf surface **(**Fig. 5b**)**, was comparable to the distribution observed on the living *A*. *thaliana* leaves **(**Fig. 5a**)**.

Furthermore, visualising bacteria on PDMS replica surfaces simplified the traditional process used for visualisation studies undertaken on living leaves. Where, neither mounting resin or cover slides were required for imaging on PDMS replica surfaces, thus, not the bacteria distribution was not disrupted. Furthermore, the PDMS replica leaf did not degrade during the bacteria visualisation studies, as is the case with living leaf samples. Thus, enabling the exact same sample to be visualised over several days for extended phyllosphere microbiology studies.

### Conclusions and future outlook

Our work has demonstrated the potential of double-casting PDMS to produce replica leaves from plants grown under all conditions, including the more challenging optimal case. Based on our observations, other reported replication protocols most likely used plants that were grown under sub-optimal (stressed) conditions. However, these protocols were not suitable for replicating *A*. *thaliana* leaves from plants grown in optimal conditions in either soil or nutrient agar. We attribute this to the leaves having a higher water content and thinner cuticular wax layer, which impeded the ability to cure the PDMS imprint. As a result, we developed a replication protocol to account for this. We observed that leaves from plants grown in either soil or culture media did not influence the topography of the leaves or the ability to replicate the leaf topography. In addition, for the first time we have highlighted the potential of replicating the delicate structure of trichomes using PDMS for both the imprint and replica. Previously, the replication of trichomes has only been possible using polyvinylsiloxane imprints, in combination with epoxy replicas.

Using microscopy and AFM imaging, we have demonstrated that our replication protocol is suitable for replicating the intricate topography of leaf surfaces, to produce replica leaves for phyllosphere microbiology studies. Furthermore, the measured surface energy of the living and PDMS replica *A*. *thaliana* leaf surfaces were comparable. To demonstrate the suitability of our replica surfaces for phyllosphere microbiology, we examined bacterial distribution using *P*. *agglomerans* 299 R::MRE-Tn7-145 and *S*. *melonis* Fr1::MRE-Tn5-145 on both living and replica *A*. *thaliana* abaxial surfaces. The distribution of bacteria observed on the PDMS replica *A*. *thaliana* abaxial leaf surfaces, were comparable to the distributions observed on the abaxial surface of living *A*. *thaliana* leaf samples.

In summary, the results presented here indicate that our replication process for producing replica leaves in PDMS is suitable for phyllosphere microbiology studies. In addition, PDMS replica leaf surfaces offer several advantages over living leaves, for example, due to negligible degradation of PDMS replica leaves phyllosphere microbiology studies can be undertaken over several days with unlimited imaging opportunities^20^. Thus, this could enable time-lapse studies of bacteria distributions to be undertaken over several days in appropriate environmental conditions (*i*.*e*. nutrient supply). In our current work, we are using these PDMS replica *A*. *thaliana* leaf surfaces to study the influence of nutrient permeability on plant-microbe interactions at a single-cell resolution.

## Supplementary information

Supplementary Information
