# Supplementary material for: Replicating Arabidopsis Model Leaf Surfaces for Phyllosphere Microbiology
Source: Sci Rep. 2019 Oct 8;9:14420. doi: 10.1038/s41598-019-50983-7 (PMC6783459; doi:10.1038/s41598-019-50983-7)
Supplement: Supplementary file 1 — Supplementary Information [file 41598_2019_50983_MOESM1_ESM.pdf]

Supplementary Information:

Replicating Arabidopsis Model Leaf Surfaces for Phyllosphere Microbiology

Rebecca Soffe,[1*] Michal Bernach,[1,2] Mitja Remus-Emsermann,[2] Volker Nock[1*]

[1] Department of Electrical and Computer Engineering, University of Canterbury, Christchurch, New Zealand

[2] School of Biological Sciences, University of Canterbury, Christchurch, New Zealand

Email: rebecca.soffe@canterbury.ac.nz; volker.nock@canterbury.ac.nz

Supplementary Information S1: Optical Microscopy Images of an *A. thaliana* Replica Leaf from a Plant Grown in Culture Media

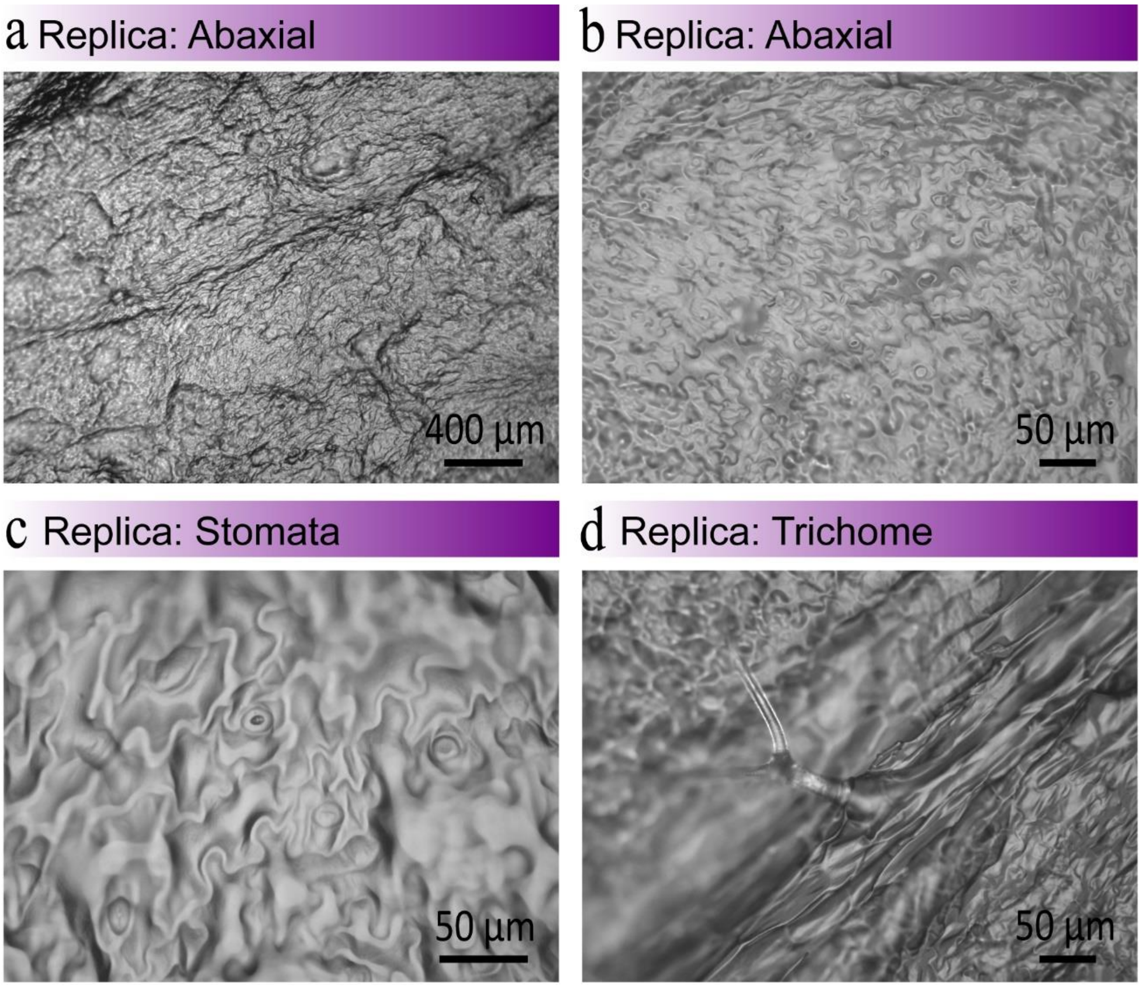

Figure S1: Optical Microscopy Images of *A. thaliana* Replica Leaves from Plants Grown in Culture Media under Optimal Conditions. (a–b) Replica abaxial leaf surfaces and magnified microstructures: (c) Stomata and (d) Trichomes.

Supplementary Information S2: Digestion of Leaf Residue Raw Data.

Weight measurements were taken to examine the potential impact of the digestion procedure to remove leaf residues. Samples were taken using a cork borer (Usbeck, Germany) with an outer diameter of 12.5 or 15.0 mm. The samples were weighed (initial) before undertaking the digestion protocol, as well as after the digestion solution, then again after being cleaned with deionised water. The samples were dried with dry nitrogen gas prior to being weighed, to remove any remaining moisture.

Table S1: Weight measurements for three samples with a diameter of 12.5 mm.

Sample	Initial (g)	Digestion (g)	Deionised Water (g)
**Sample 1**	0.2724	0.2727	0.2724
**Sample 2**	0.2799	0.2799	0.2799
**Sample 3**	0.3157	0.3185	0.3159

Table S2: Weight measurements for three samples with a diameter of 15.0 mm.

Sample	Initial (g)	Digestion (g)	Deionised Water (g)
**Sample 1**	0.3234	0.3242	0.3234
**Sample 2**	0.2873	0.2874	0.2876
**Sample 3**	0.3152	0.3154	0.3152

Supplementary Information S3: Large Area AFM Image of Stoma Imprint

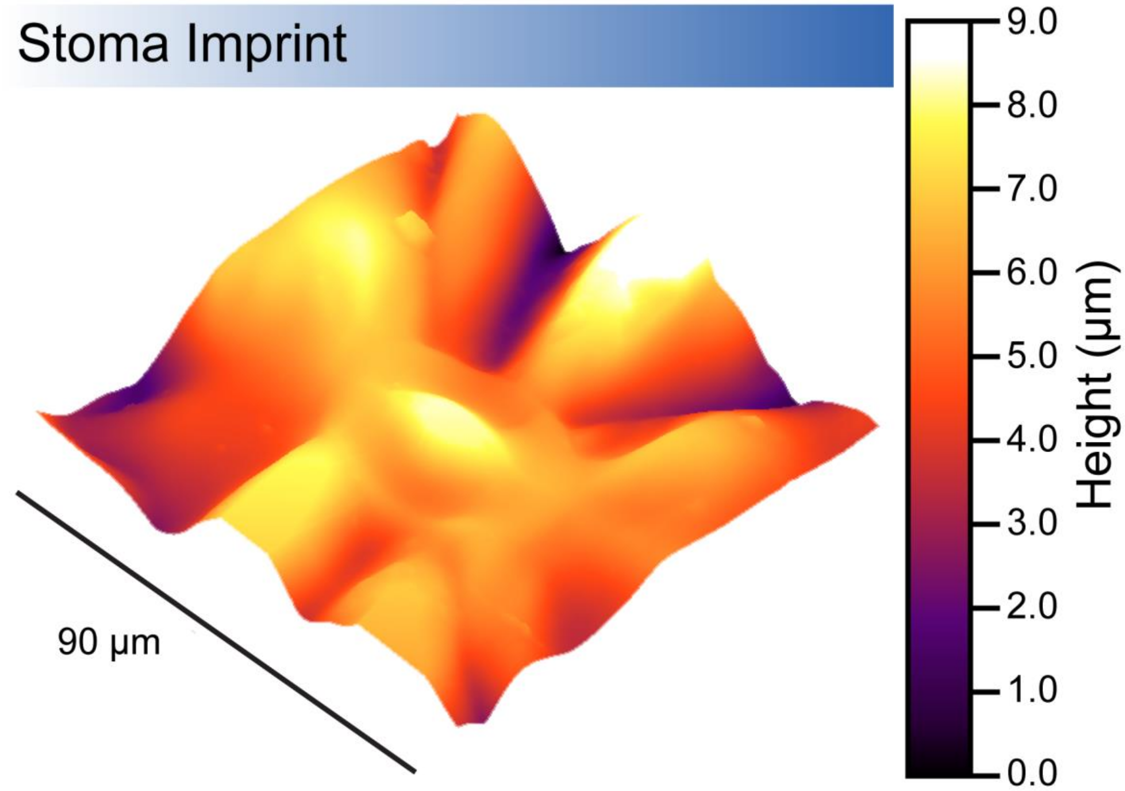

Figure S2: Large Area AFM Image of *A. thaliana* Stoma Imprint.

Supplementary Information S4: Bacterium *P. agglomerans* 299R::MRE-Tn7-145 Visualised on Living and Replica *A. thaliana* Leaves

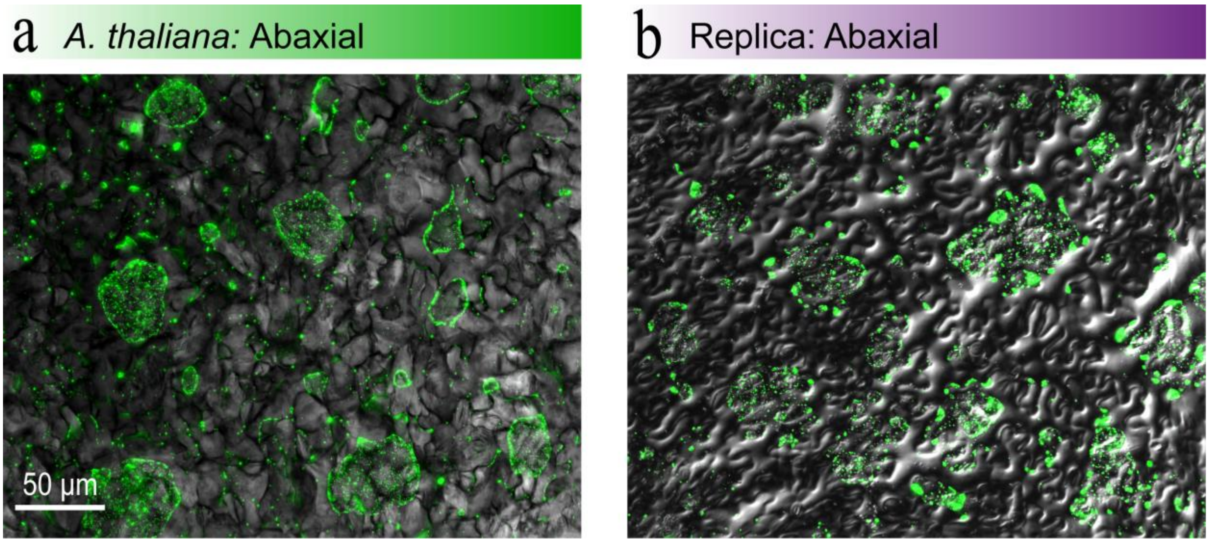

Figure S3: Bacterial Distribution on Abaxial Surfaces. The bacterium P. agglomerans 299R::MRE-Tn7-145 (green) with an $OD_{600\ nm}$ of 0.7 visualised on (a) living A. thaliana and (b) PDMS replica leaf abaxial surfaces.
